# Origin of the mechanism of phenotypic plasticity in satyrid butterfly eyespots

**DOI:** 10.7554/eLife.49544

**Published:** 2020-02-11

**Authors:** Shivam Bhardwaj, Lim Si-Hui Jolander, Markus R Wenk, Jeffrey C Oliver, H Frederik Nijhout, Antonia Monteiro

**Affiliations:** 1 Department of Biological SciencesNational University of SingaporeSingaporeSingapore; 2 Department of BiochemistryNational University of SingaporeSingaporeSingapore; 3 Office of Digital Innovation & StewardshipUniversity of ArizonaTucsonUnited States; 4 Department of BiologyDuke UniversityDurhamUnited States; 5 Yale-NUS CollegeSingaporeSingapore; University of MichiganUnited States; University of California, Los AngelesUnited States

**Keywords:** phenotypic plasticity, 20E, ecdysone, seasonal polyphenism, eyespot size, lepidoptera, Other

## Abstract

Plasticity is often regarded as a derived adaptation to help organisms survive in variable but predictable environments, however, we currently lack a rigorous, mechanistic examination of how plasticity evolves in a large comparative framework. Here, we show that phenotypic plasticity in eyespot size in response to environmental temperature observed in *Bicyclus anynana s*atyrid butterflies is a complex derived adaptation of this lineage. By reconstructing the evolution of known physiological and molecular components of eyespot size plasticity in a comparative framework, we showed that 20E titer plasticity in response to temperature is a pre-adaptation shared by all butterfly species examined, whereas expression of EcR in eyespot centers, and eyespot sensitivity to 20E, are both derived traits found only in a subset of species with eyespots.

## eLife digest

A well-known family of butterflies have circular patterns on their wings that look like eyes. These eye-like markings help deflect predators away from the butterfly’s body so they attack the outer edges of their wings. However, in certain seasons, such as the dry season in Africa, the best way for this family to survive is by not drawing any attention to their bodies. Thus, butterflies born during this season shrink the size of their eyespots so they can hide among the dry leaves.

How this family of butterflies are able to change the size of these eye-like spots has only been studied in the species *Bicyclus anynana*. During development low temperatures, which signify the beginning of the dry season, reduce the amount of a hormone called 20E circulating in the blood of this species. This changes the behavior of hormone-sensitive cells in the eyespots making them smaller in size. But it remains unclear how *B. anynana* evolved this remarkable tactic and whether its relatives have similar abilities.

Now, Bhardwaj et al. show that *B. anynana* is the only one of its relatives that can amend the size of its eyespots in response to temperature changes. In the experiments, 13 different species of butterflies, mostly from the family that has eyespots, were developed under two different temperatures. Low temperatures caused 20E hormone levels to decrease in all 13 species. However, most of these species did not develop smaller eyespots in response to this temperature change. This includes species that are known to have larger and smaller eyespots depending on the season.

Like *B. anynana,* four of the species studied have receptors for the 20E hormone at the center of their eyespots. However, changing 20E hormone levels in these species did not reduce eyespot size.

These results show that although temperature changes alter hormone levels in a number of species, only *B. anynana* have taken advantage of this mechanism to regulate eyespot size. In addition, Bhardwaj et al. found that this unique mechanism evolved from several genetic changes over millions of years. Other species likely use other environmental cues to trigger seasonal changes in the size of their eyespots.

## Introduction

There are two disparate views regarding phenotypic plasticity. One regards plasticity as a derived adaptation to help organisms survive in variable environments (Bradshaw, 1965; De Jong, 2005) while the other views plasticity as the outcome of flexible, non-canalized, developmental processes, ancestrally present in most organisms, that helps them colonize or adapt to novel environments, a type of pre-adaptation (Newman and Müller, 2000; West-Eberhard, 2003; Pigliucci, 2005; Laland et al., 2014). While both views of plasticity are likely valid, they both currently lack a rigorous, mechanistic examination of ancestral and derived states and direction of change (Bradshaw, 1965). Furthermore, the two views on phenotypic plasticity articulated above, as an adaptation or a pre-adaptation, require either that plasticity evolves under natural selection or that it is ancestral and widespread and facilitates adaptation. Several case studies have been documented in support of the first (Moran, 1992; Wund et al., 2008; Standen et al., 2014) and second evolutionary scenarios (Kiontke and Fitch, 2010; Hiyama et al., 2012) but to date, almost nothing is known about how the plastic responses underlying both scenarios originated and evolved at the proximate, mechanistic level. Details of how plasticity evolves, and whether or not it is widespread and ancestral to a group of species, regardless of their current living environments, may also help discriminate between plasticity being a facilitator or a consequence of organismal adaptation.

Here we focus our investigation on the mechanistic evolution of an adaptive seasonal polyphenism where environmental cues experienced during development alter adult phenotypes to make them fit different seasonal recurrent environments, a highly evolved form of phenotypic plasticity. We use dramatic seasonal variation in the size of *B. anynana* wing eyespot patterns as our case study. *Bicyclus* species live throughout dry and wet seasons in Africa, where eyespots of different sizes serve different ecological roles (Brakefield and Reitsma, 1991; Brakefield et al., 1996). In the hot wet season, the large exposed ventral eyespots help deflect attacks of invertebrate predators, or naïve vertebrate predators towards the wing margins (Lyytinen et al., 2004; Olofsson et al., 2010; Prudic et al., 2015), whereas in the cool dry season the smaller eyespots help in camouflage against vertebrate predation (Lyytinen et al., 2004).

Because eyespot size plasticity in *B. anynana* is sufficiently well understood at the molecular level, this species becomes an ideal springboard for a comparative approach that addresses the mechanistic evolution of this form of plasticity across a phylogeny. Eyespot size plasticity in *B. anynana* is mostly controlled by temperature, which leads to variable titers of the hormone 20-hydroxyecdysone (20E) at the wandering (Wr) stage of larval development (Monteiro et al., 2015). Manipulations of 20E signaling alone, at that time in development, are sufficient to modify eyespot size (Monteiro et al., 2015). This is because these central cells express the 20E receptor, Ecdysone Receptor (EcR), and upon sufficient 20E signaling, the active 20E-EcR complex is able to interact with yet unknown downstream genes to make these central cells divide and produce a larger central group of signaling cells (Bhardwaj et al., 2017) and ultimately a larger eyespot. Given knowledge of how eyespot size plasticity functions in this species, we sought to investigate how this system of temperature sensitivity evolved by performing a comparative study across nymphalid butterflies, with and without eyespots.

Eyespots originated once within the nymphalid family, about 85 mya, likely from pre-existing simple spots of a single color (Oliver et al., 2012; Oliver et al., 2014) but it is unclear whether size plasticity in response to temperature evolved before or after the origin of eyespots. If eyespot or spot size plasticity is an ancestral pre-adaptation, it is possible that even species of butterflies that do not experience seasonal environments (such as those living near the equator), might have the ability to develop different eyespot or spot sizes when reared at different temperatures under experimental conditions. Alternatively, if eyespot size plasticity is an evolved adaptation, used exclusively by species living in seasonal environments, then only these species should exhibit plasticity.

## Results

**Figure 1.**
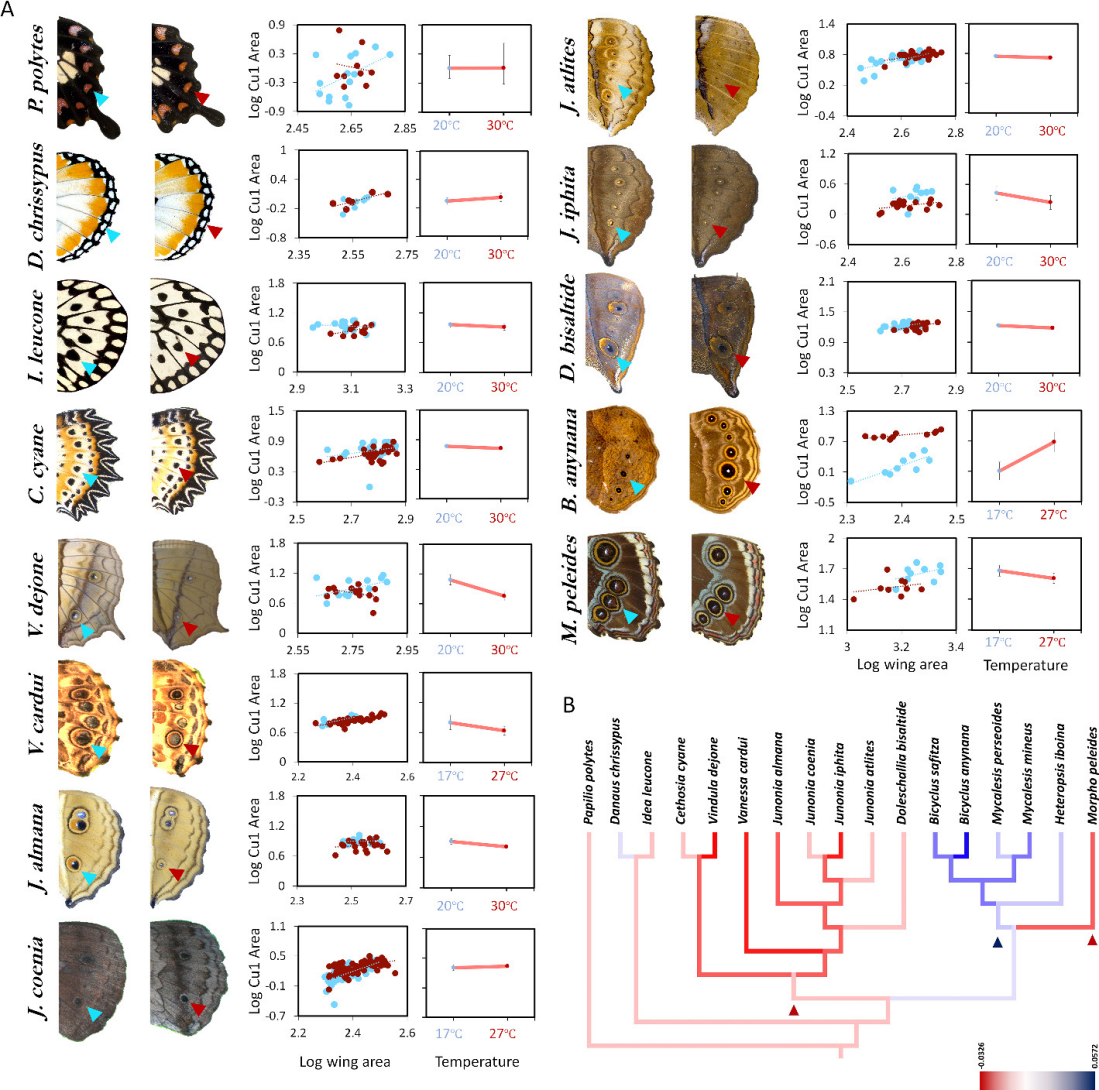
Eyespot/spot size plasticity is widespread across butterfly lineages but the response to rearing temperature has different norms of reaction across species. (**A**) Size of hindwing ventral Cu1 eyespots (arrowheads). Thirteen species of butterflies were reared at two different rearing temperatures. Eyespot size corrected for wing size is plotted for two different temperatures (low temperature 17°C or 20°C is marked with blue symbols, while high temperature of 27°C or 30°C is marked with red symbols). Error bars represent 95% CI of means. (**B**) Mapping origins of eyespot size plasticity via maximum parsimony phylogenetic analysis suggests three independent origins for two different patterns of plasticity in the lineage with eyespots (eyespot size decreases with increasing temperatures: red lineages, and eyespot size increases with increasing temperature: blue lineage). The lineage leading to Satyrid butterflies gained a positive response to plasticity (blue arrowhead), whereas most other Nymphalids had either no response, or limited negative plasticity response (red arrowhead). Figure 1—source data 1.Supporting details for Figure 1.Table S1 - Species reared for comparative morphometrics, gene expression and hormonal measurements. Table S2 - F statistics, p-values from analysis of covariance for differences in Cu1 eyespot size between rearing temperatures (fixed factor) and assigned character state for phylogenetic analysis.

To test these hypotheses and to examine how plasticity in *B. anynana* evolved, we reared twelve species from different nymphalid sub-families, and from tropical, or sub-tropical regions, plus one outgroup papilionid species (Figure 1—source data 1—Table S1) at two different temperatures, separated by 10°C, and measured spot and eyespot size plasticity in adult females. Three different types of reaction norm to rearing temperature were observed across species (Figure 1A). Five species showed no significant difference in hindwing (HW) Cu1 spot/eyespot size when reared across two temperatures and were deemed not plastic. Most species showed a decrease in spot/eyespot size with an increase in temperature and had a negative slope in their reaction norms. *B. anynana* was the only species which displayed a positive slope in its reaction norm, where eyespot size increased with temperature (Figure 1—source data—Table S2). Ancestral character state reconstructions for the slope of these reaction norms suggested that eyespot size plasticity of any form is a derived trait within nymphalids, with three possible independent origins. Ancestral species of nymphalids lacked plasticity, whereas there were one or two independent origins of a negative response of eyespot size to increasing temperature and a separate origin of the opposing pattern of plasticity in ventral HW eyespot size in the Satyrid lineage, such as those leading to *B. anynana* (Figure 1B).

**Figure 1—figure supplement 1.**
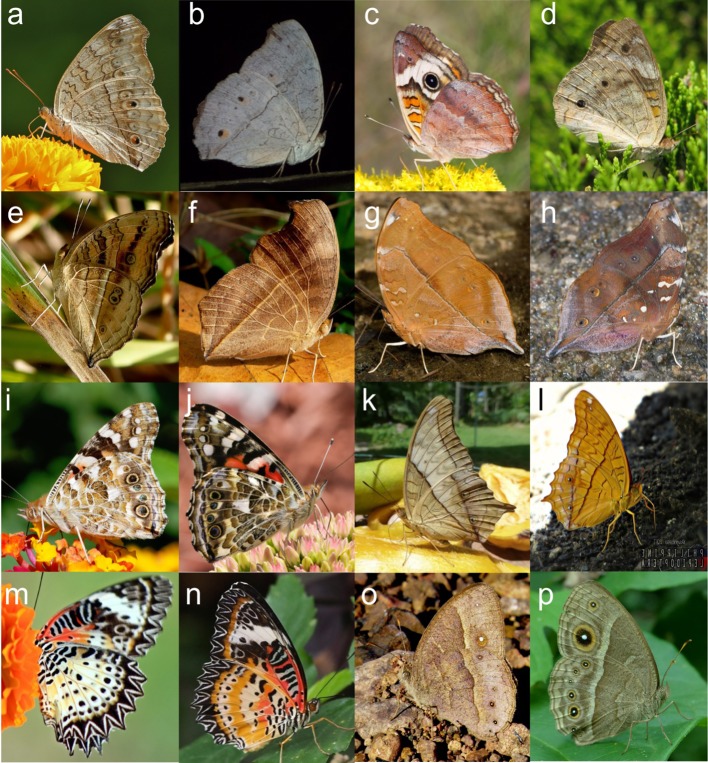
Phenotypic plasticity in wing patterns is observed across a wide variety of species in wild.

**Figure 1—figure supplement 2.**
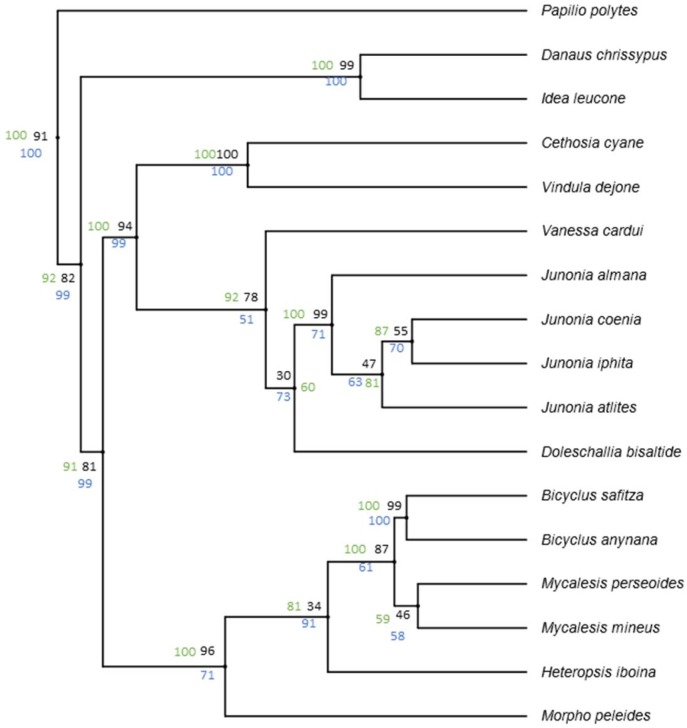
Data from Wahlberg et al. (2009) show high support value for the basal nodes across different analysis methods.

**Figure 1—figure supplement 3.**
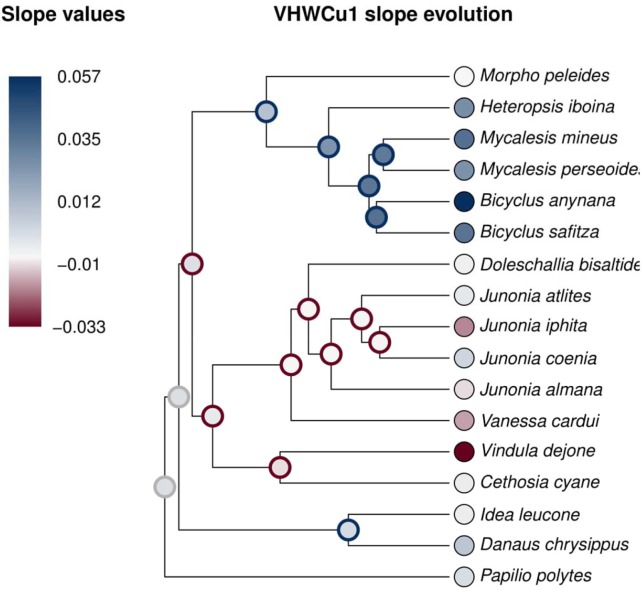
Ancestral state reconstruction using maximum likelihood models suggest that positive directionality of plasticity is a derived trait in Satyrid butterflies. Ancestral states are significantly positive (blue outline), significantly negative (red outline), or not significantly different from zero (gray outline).

**Figure 1—figure supplement 4.**
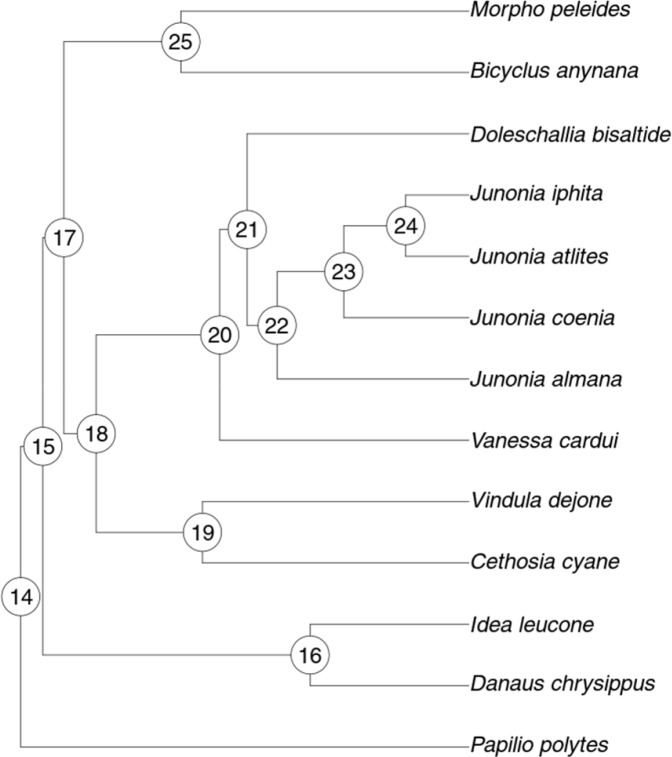
Tree used for ancestral state hypotheses tests. See text and Figure 4—source data 1 for explanation of node numbers.

**Figure 2.**
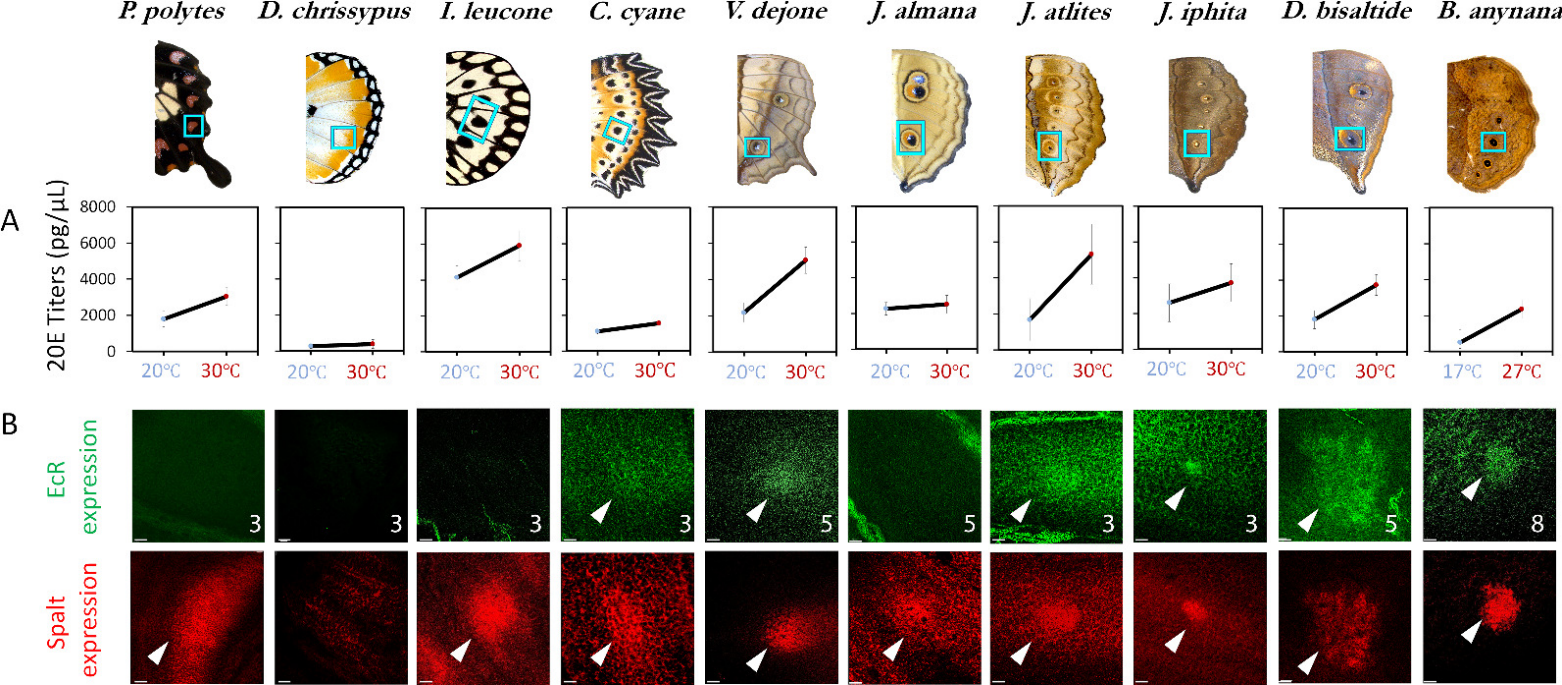
20E titers increase with rearing temperature across most species but EcR expression is only found in a subset of nymphalids with eyespots. (**A**) 20E titers increase with an increase in rearing temperature across most species. This trait is ancestral in nature, with a likely origin before the origin of eyespots. (**B**) EcR is absent in simple spots, but present in the future eyespot centers of most of the species investigated (N ≥ 3 for each immunostaining: numbers in superscript represent sample size; Scale bars,10µm). Figure 2—source data 1.F statistics, p-values from analysis of covariance for differences in 20E hormone titers between rearing temperatures (fixed factor) and assigned character states for phylogenetic analysis.

To investigate the molecular basis for how these distinct patterns of plasticity differed from that of *B. anynana* we compared 20E titers and EcR expression across species using female data, and focused exclusively on examining data for the critical period of development that was previously discovered for the regulation of Cu1 eyespot size plasticity in ventral hindwings of *B. anynana*, for example the wandering stage of larval development. 20E titers at the wandering stage were consistently higher at the higher rearing temperature across all butterflies (Figure 2A) (Figure 2—source data 1), suggesting that 20E titer plasticity in response to temperature is an ancestral trait shared across these butterflies. EcR expression at the wandering stage was absent in species with no central wing spots (e.g., *Danaus*), it was absent from spot centers in species with simple spots (e.g., *Papilio* and *Idea*), but was present in the eyespot central cells across all other species investigated, with a few exceptions (*Junonia coenia* (Koch et al., 2003) and *Junonia almana*) (Figure 2B). Species with no EcR staining in spots still had EcR expression in the large polyploid nuclei that make up the peripodial membrane that wraps around each larval wing (Figure 2—figure supplement 1). In contrast, the spot and eyespot focal marker gene Spalt, was present in all species with eyespots or with simpler spots, as previously reported (Oliver et al., 2012; Stoehr et al., 2013). The absence of EcR expression in *J coenia* eyespots is restricted to the wandering stage of development, as EcR is expressed in the eyespots of this species in later stages of development (Koch et al., 2003). The EcR expression data overall suggests that EcR localization in eyespots, at the wandering stages of development, is a derived trait, present only in species with eyespots. Some of these eyespotted species subsequently lost EcR expression at this stage of development, a phenomenon previously reported for forewing Cu1 ventral eyespots in *B. anynana* females (Monteiro et al., 2015).

**Figure 2—figure supplement 1.**
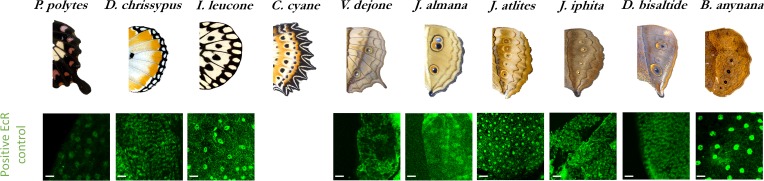
EcR expression as a positive control in peripodial membrane nuclei acrossspecies. (N ≥ 3 for each species, Scale bars, 10µm).

**Figure 2—figure supplement 2.**
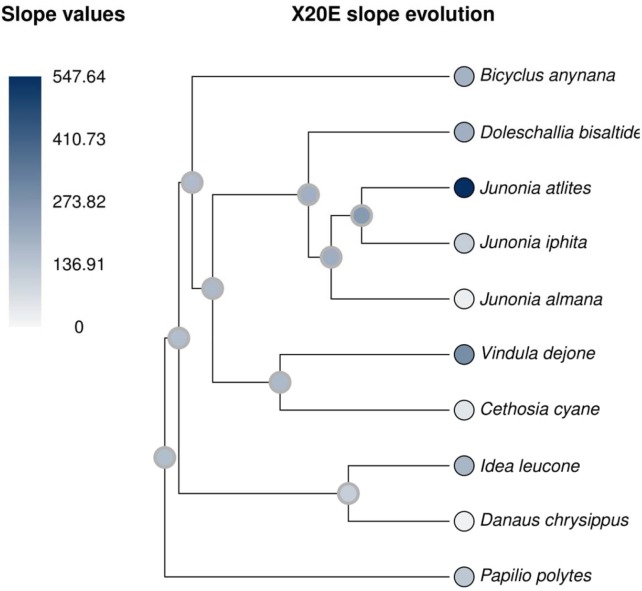
20E titers increase with increasing temperatures as an ancestral trait, present at the basal node of Nymphalids and outgroups. All ancestral states are not significantly different from zero (gray outline).

**Figure 3.**
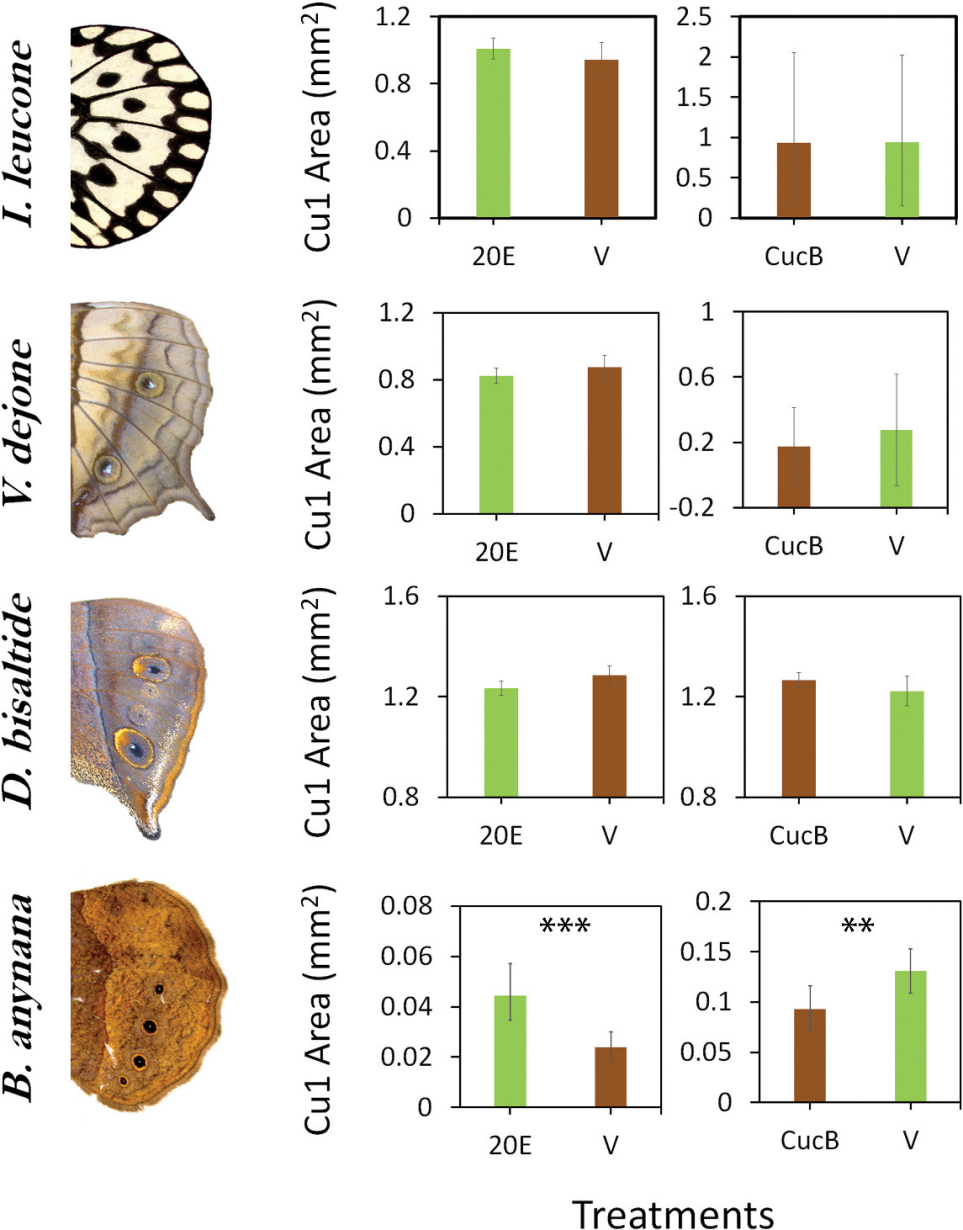
Sensitivity of eyespots to EcR-mediated signaling evolved in the lineage leading to *B. anynana* butterflies. Four species of butterflies were injected with 20E hormones or EcR antagonists (CucB) during the wandering (Wr) stage. Control larvae were injected with an equal volume solution of saline vehicle (V). While *Idea leuconoe, Vindula dejone and Doleschallia bisaltide* are not sensitive to either of the hormone signal manipulations, *B. anynana* shows sensitivity towards both 20E and CucB. Error bars represent 95% CI of means. Significant differences between treatments are represented by asterisks: **, p<0.01, ***, p<0.001. Figure 3—source data 1.Mean body weight of wandering larvae, hemolymph volume and natural 20E titers at two different rearing temperatures; 20E and CucB injection volume.

**Figure 4.**
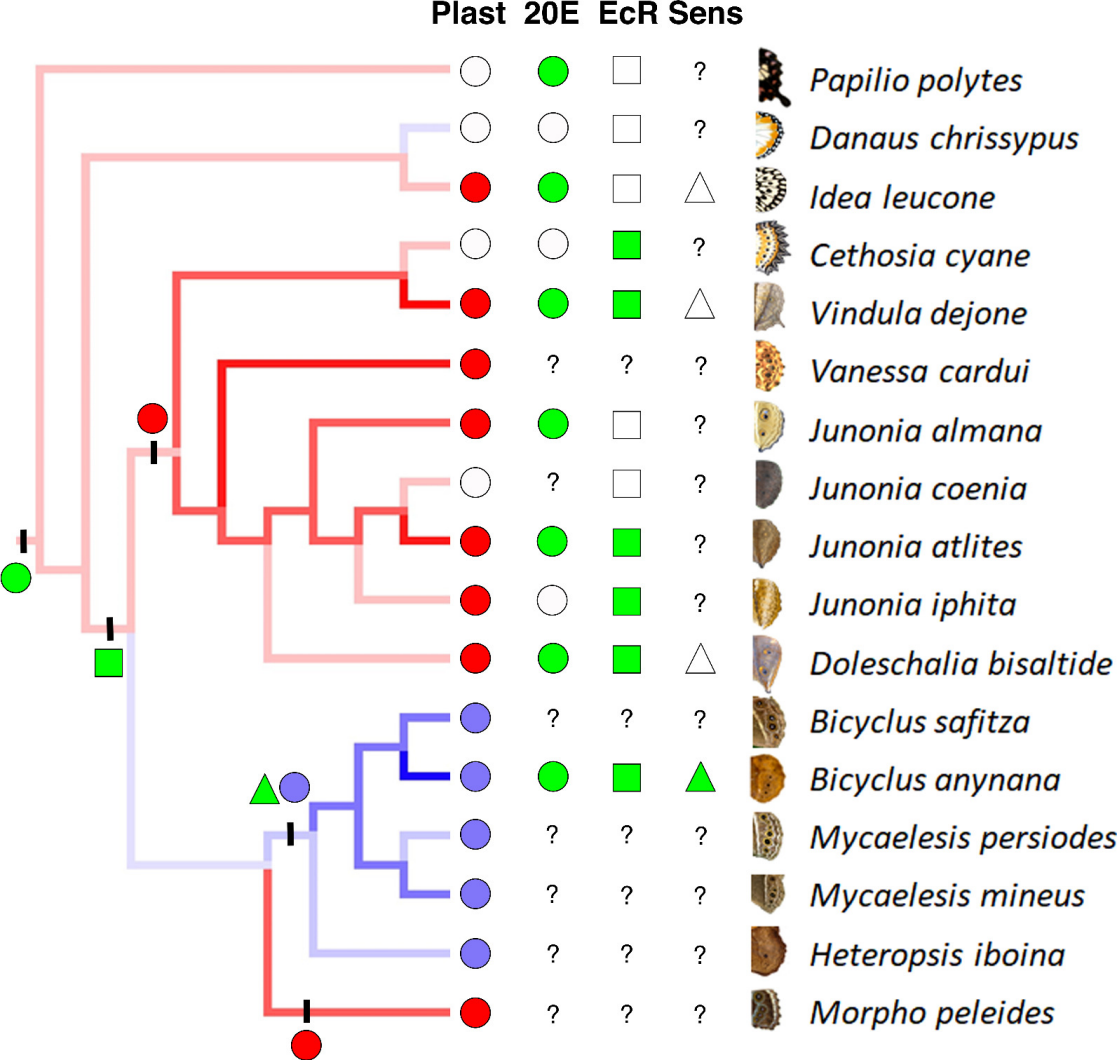
Phenotypic plasticity as a complex trait evolved gradually. Phylogenetic analysis suggests three independent origins for two different patterns of eyespot size plasticity (eyespot size decreases with increasing temperatures: red lineages and red circles, and eyespot size increases with increasing temperature: blue lineage and blue circles). Empty circles represent a lack of plastic response. Green circles (character state 1) represent high 20E titers with increasing temperature, while white circles (character state 0) represent no significant difference in titers at two developmental temperatures. Green squares represent presence of EcR in eyespots, while white squares represent its absence. EcR expression in eyespots is inferred to have originated concurrently with the origin of eyespots, about 85 Mya, and subsequently lost in a few nymphalid lineages. Green triangles represent sensitivity towards 20E (character state 1), while white triangles represent absence of sensitivity (character state 0). Question marks represent missing data points. Circles, square and triangle on left with vertical bars represent respective estimated evolution of eyespot size plasticity (red and blue circles), 20E titer plasticity (green circle), EcR expression in eyespots (green square) and sensitivity towards 20E (green triangle). Alternative models using Maximum Likelihood reach similar conclusions (Supplementary Information: Figure 1—figure supplement 2, 3, Figure 4—source data 1). H. iboina image copyright of David.C. Lees, Cambridge University Department of Zoology. Figure 4—source data 1.Results of likelihood ratio tests and AIC comparisons.See Figure 1—figure supplement 1 for node identities.

Finally, to test whether eyespots expressing EcR are size-regulated by 20E we manipulated 20E levels and EcR-mediated signaling directly, focusing again, exclusively on the wandering stages of development, previously shown to be the critical hormone-sensitive stage in *B. anynana*. Functional experiments were performed in four species of butterflies from different Nymphalid subfamilies, *Idea leuconoe* (Danainae), a control outgroup danainae with no EcR expression in its black spots, *Vindula dejone* (Nymphalinae)*, Doleschallia bisaltide* (Nymphalinae), and *B. anynana* (Satyrinae), the latter three displaying EcR expression in their eyespot centers. Our prediction would be that *Idea* should not respond to 20E signaling at all, given the lack of the receptor in its spots, and that increases in 20E signaling at low temperature might cause the eyespots of *Vindula* and *Doleschalia* to become smaller but those of *B. anynana* to become larger, whereas decreases of 20E signaling at high temperature might cause the eyespots of the first two species to become larger but smaller in *B. anynana*. Injections of 20E into female wanderers reared at low temperature (and with lower 20E titers) and of an EcR antagonist, CucB, into female wanderers reared at high temperature (and with higher 20E titers), showed no response across the first three species, whereas eyespot size significantly increased with 20E injections and decreased with antagonist injections in *B. anynana* (Figure 3). These data indicate that only the eyespots of *B. anynana* are sensitive to 20E signaling at the wandering stage, within the natural range of titers displayed by these species. This sensitivity is a derived trait potentially restricted to the satyrid sub-family within nymphalids (Figure 4).

## Discussion

While multiple reports have focused on the role of hormones as mediators of developmental plasticity in a variety of traits (Emlen and Nijhout, 1999; Dai et al., 2001; Gotoh et al., 2011; Gotoh et al., 2014), the physiological and developmental details of how a fully functional plastic trait evolves during the course of evolution were still obscure. Here, we identified the approximate evolutionary origins of individual components of a plastic response of eyespot size in response to temperature and discovered this plastic response to be a complex trait that evolved gradually via changes to different molecular components. Our work showed that the evolution of plasticity in hormone titers, the evolution of hormone receptor expression in the trait, and the evolution of eyespot sensitivity to these hormones all took place at different stages of nymphalid diversification (Figure 4).

An increase in eyespot size in response to temperature appears to be restricted to satyrid butterflies, and is a derived response within nymphalids. Plasticity in eyespot size in butterflies had been primarily documented in satyrid butterflies such as *Melanitis leda* and several *Bicyclus* species (Brakefield, 1987; Roskam and Brakefield, 1999; van Bergen et al., 2017) where size was always found to increase with rearing temperature. Most of the reared species of nymphalids and the papilionid species showed a slight decrease in eyespot/spot size with an increase in temperature, while some species showed no plasticity at all. This decrease in eyespot size with increasing temperature may simply reflect non-adaptive variation from a poorly canalized system. In addition, satyrid butterflies, but none of the other species, used the 20E asymmetry to regulate the size of their eyespots in a novel way. This was enabled by the prior recruitment of EcR to the eyespot central cells perhaps concurrently with eyespot origins (Figure 4). These central signaling cells play an important role in determining eyespot size at the wandering stages of development (Monteiro et al., 1994). Some species, such as *Junonia coenia*, retain expression of EcR in eyespots but only at other stages of wing development (Koch et al., 2003). Finally, the active 20E-EcR complexes increase eyespot size in *B. anynana* but not in other species with similar EcR expression in their eyespot centers. The ability of 20E to promote localized patterns of cell division might have evolved in the lineage leading to *B. anynana* alone (Bhardwaj et al., 2017).

Eyespot size plasticity in connection with wet and dry seasonal forms is widely conserved across the sub-family Nymphalinae (Clarke, 2017) but our results suggest that different mechanisms may have evolved to regulate eyespot size plasticity in these lineages. Our controlled rearing experiments showed that all nymphalinae (*Vanessa cardui, Junonia almana, J. coenia, J. atlites, J. iphita* and *Doleschallia bisaltide*) produced only small changes in the size of the their Cu1 eyespots in response to rearing temperature, and these were in the opposite direction to those observed in *B. anynana*. Other environmental factors might cue and regulate these species' seasonal morphs (Figure 1—figure supplement 1), perhaps cues that better predict the arrival of the seasons where these butterflies have evolved. Investigations at the proximate level will be required to correctly establish the environmental cues that induce seasonal forms in these other butterfly species. In addition, a broader temporal investigation of the critical developmental periods of environmental sensitivity, followed by investigations of hormonal sensitivity at those critical periods, will need to be performed for these other species in future. Future investigations can also expand the evolution of plasticity to eyespots not examined here. For now, we uncover phenotypic plasticity in ventral (Cu1) eyespot size in *B. anynana* hindwings as a complex, step-wise adaptation to seasonal environments cued by temperature that required very specific mutations to evolve. This work also serves as a warning that if many forms of adaptive plasticity are as specific and hard to evolve as the one documented in *B. anynana*, these exquisite adaptations to specific predictable fluctuating environments may in fact, lend the species vulnerable to extinction under unpredictable climate change, as previously noted (Oostra et al., 2018).

## Materials and methods

**Key resources table:** 

Reagent type (species) or resource	Designation	Source or reference	Identifiers	Additional information
Strain, strain background (*Butterflies, females*)	*Junonia atlites; Junonia coenia; Junonia iphita*; *Junonia almana*; *Doleschallia bisaltide*; *Vanessa cardui*; *Vindula dejone*; *Cethosia cynae*; *Bicyclus anynana*; *Morpho peleides*; *Danaus chryssipus*; *Idea leuconoe*; *Papilio polytes*	Penang Butterfly Farm, Malaysia; Duke University; Yale University; National University of Singapore		
Antibody	EcR common isoform, *Manduca sexta*	DSHB		1:10
Antibody	Spalt, Primary antibody, Guinea pig	Stoehr et al., 2013		1:20000
Antibody	AlexaFlour 488 green Goat anti-mouse secondary antibody	Molecular Probes	Cat# A-11001, RRID:AB_2534069	1:800
Antibody	Goat anti-Guinea pig secondary antibody	Molecular Probes	Cat# A-11076, RRID:AB_141930	1:800
Chemical compound, drug	20-Hydroxyecdysone (20E)	Sigma-Aldrich	Cat# H5142	Lot # 060M1390V
Chemical compound, drug	Cucurbitacin B (CucB)	Sigma–Aldrich	Cat# C8499	Lot # 035M47104V
Software, algorithm	Imaris v8.64	(ImarisXT, Bitplane AG)		
Software, algorithm	Rphylopars	(Goolsby et al., 2017)		
Software, algorithm	ape	(Paradis et al., 2004)		
Software, algorithm	packages for R	(R Development Core Team, 2018)		

### Butterfly husbandry

All species were reared at two temperatures separated by 10 degrees, at 70 or 80% RH and at 12:12 hr light: dark cycle. The only exception was *Junonia coenia,* which was reared at 16:8 hr light: dark cycle and 80% RH. *B. anynana* was reared in climate control chambers in Singapore, at 17°C and 27°C, at 80% RH. *Vanessa cardui*, and *Morpho peleide*s were reared in climate control chambers at Yale University, New Haven at 17°C and 27°C, and at 80% RH. *Junonia coenia* was reared at 20° and 30°C at Duke University. All other species of butterflies were reared at Entopia (formerly, Penang Butterfly Farm, Penang, Malaysia) in temperature-controlled chambers (PT2499 Incubator, Exoreptiles, Malaysia) at 20°C and 30°C, and at 70% RH. Humidity in these latter chambers was monitored using (PT2470 Hygrometer, Exoreptiles, Malaysia) and EL-USB-2 data loggers (Lascar Electronics, PA 16505, USA).

Four hours after emergence, butterflies were captured and frozen in glassine envelopes at −20°C. All larvae in this experiment were sexed during larval or pupal stages and only females were used for analysis. Wings were carefully dissected and imaged using a Leica upright microscope. Wing images were processed in ImageJ, where area and eyespot size were measured using selection tools.

Additional data for Nymphalid species not reared in Figure 1—source data 1—Table S1 but mentioned in Figure 1, Figure 4, Figure 1—figure supplement 2 and Figure 1—figure supplement 3 were extracted and analyzed from van Bergen et al. (2017). These species were reared at 21°C and 27°C, and slopes were corrected for a temperature difference of 6°C, instead of usual 10°C for other species used in this study.

### Hemolymph collection

Previous studies in *B. anynana* have pointed to the wandering (Wr) stage as the critical temperature sensitive stage for determination of ventral hindwing eyespot size (Monteiro et al., 2015). Time lapse photographs of larval development were captured every 15 min using a RICOH camera to determine the beginning of the Wr stage across all species. Initiation of Wr stage is marked by the larvae stopping to feed, purging their gut, and starting to wander away from the food and looking for a place to pupate. Using Hamilton syringes, 20 µL of hemolymph, were extracted from each larvae at ~70% development in Wr stage (15 hr after Wr started for animals reared at 30°C, and 25 hr for animals reared at 20°C). Extracted hemolymph was then dissolved in freshly prepared 90 µl methanol + 90 µl isooctane and stored at −20°C until hormone extraction (Bhardwaj et al., 2017) .

### Wing tissue collection

Larval wing discs were dissected from Wr stage larvae at 27°C or 30°C and stored in fix buffer until further processing at 4°C. These were later stained for EcR expression using a primary antibody 15F1 (DSHB) raised against a *Manduca sexta* EcR peptide shared across all isoforms of EcR. AlexaFlour 488 green Goat anti-mouse (Thermo Fisher Scientific Cat# A-11001, RRID:AB_2534069) was used as secondary antibody at a dilution of 1:800 for EcR stains. Primary antibodies against Spalt, a previously published (Stoehr et al., 2013) nuclear marker for spots and eyespots, was used at a dilution of 1:20000, supported with Goat anti-Guinea pig secondary antibody (Molecular Probes Cat# A-11076, RRID:AB_141930; at a dilution of 1:800), as a location marker for putative eyespots/spots in the larval wings. Serial optical sections of the Cu1 eyespot wing sector were imaged using LSM510 Meta, to distinguish between dorsal and ventral surfaces. Specific slices were obtained from raw images using Imaris v8.64 (ImarisXT, Bitplane AG, software available at http://bitplane.com. *Junonia coenia* EcR data were taken from Koch et al. (2003).

### 20E and antagonist injections

Four species of butterflies, *Idea leuconoe, Vindula dejone, Doleschallia bisaltide,* and *B. anynana,* were injected with 20E or CucB during the Wr stage. Injections were made at ~50% development of Wr stage (12–14 hr at 30°C, 18–22 hr at 20°C; For *B.anynana,* rearing were done at 27°C and 17°C, respectively). Average body weights of wandering larvae and total hemolymph present were calculated for each species, and used to calculate naturally circulating 20E levels in vivo. A gradient of different concentrations of 20E and CucB were used for pilot experiments. Maximum concentrations of 20E, which did not surpass the natural levels, and of CucB, which did not cause mortality or pupation defects, were used for injections and are summarized in the table below. 20E and CucB were dissolved in 10% EtOH to make working solution for injections. Equal volume injections of Vehicle (10% EtOH in Saline) injections were done as controls (Figure 3—source data 1). After injections, animals were reared at their regular rearing temperature (17°C for *B.anynana*, 20°C for other 20E injected animals and 27°C for *B.anynana*, 30°C for CucB injected animals) until emergence as adults. After emergence, the wings were dissected, imaged, and scored for further analysis.

### Statistical analysis

All wing and eyespot data were log10 transformed to ensure linearity of wing size with eyespot size for purposes of allometric scaling and regression analysis, and to be able to compare slopes across species with different eyespot sizes and wing sizes. Univariate ANCOVAs were performed using hindwing Cu1 eyespot area as the main variable, hindwing area as a covariate, and rearing temperature as a fixed factor in SPSS v21. Graphs were plotted in Microsoft Office 2016 for Mac. Slopes for plasticity of eyespot size and 20E titers were measured using the expression:

$$Slope=\frac{\left(Value\;at\;high\;temperature-Value\;at\;low\;temperature\right)}{Difference\;in\;rearing\;temperature\;(10^{\circ }C)}$$

Using reverse transformed data for eyespot size, and untreated values for hormone titers.

### Phylogenetic analysis

Patterns of plasticity in eyespot size were categorised in distinct groups based on positive, negative, or slopes undistinguishable from zero when eyespot size was plotted against temperature. Using a pruned version of a larger phylogenetic tree for all nymphalid genera (Wahlberg et al., 2009; Oliver, 2013), ancestral trait reconstructions were performed, and evolution of the reaction norm slopes was mapped using maximum parsimony in Mesquite. Similar analyses were performed using data obtained for hormone titer plasticity where species were categorized into two categories – those with a positive slope or a zero slope, and data for presence or absence of EcR expression, and 20E-EcR signaling affecting eyespot size.

We also evaluated several hypotheses concerning the evolution of relevant traits with likelihood ratio tests (LRT) and Akaike Information Criteria (AIC). For all analyses, specific ancestral nodes of interest were 'fixed' for a particular state and the resultant maximum likelihood score was used for LRT and AIC comparisons (Oliver et al., 2012). We performed four tests in all, investigating (1) whether the most recent common ancestor (MRCA) to all butterflies (node 14 in Figure 1—figure supplement 4) had plasticity in spot and eyespot size or not; (2) whether the MRCA to all butterfly species with eyespots (node 17) had plasticity in eyespot size or not; (3) whether the MRCA to all butterflies (node 14) had positive hormone titre plasticity or not; and (4) whether the MRCA to all butterfly species with eyespots (node 17) expressed EcR in the locations of future spots / eyespots or not. For tests of eyespot size plasticity, we used a three-state coding scheme: positive size plasticity, negative size plasticity, and no plasticity. Character states were scored based on the sign of the slope of the reaction norm; species with reaction norms that were not significantly different from zero were scored as having no temperature-dependent plasticity in eyespot size (Figure 1—source data 1—Table S2). Tests on positive hormone titer plasticty and EcR expression used characters coded as binary states. For AIC comparisons, we used the correction for small sample sizes (AICc) and evaluated models based on the AICc weight, *w_i_* = e^((min(AICc – AICc)/2)^. Models were considered significantly different if they differed by 2 or more log-likelihood units or the AICc weight was less than 0.2.

For all comparisons, there was little significant support for one hypothesis over another (Figure 4—source data 1). In tests on the origin of eyespot size plasticity, both the MRCA to all butterflies and the MRCA to all butterflies with eyespots had slightly better likelihood and AICc scores for being non-plastic than being plastic. Positive hormone titer plasticity in the MRCA to all butterflies had more support than a non-plastic MRCA, although the difference in likelihoods and AICc was not significant. Finally, the absence of EcR expression in the MRCA of all eyespot-bearing butterflies had higher likelihood and AICc scores than a model in which the MRCA did express EcR in future spot / eyespot centers. The absence of significant support for one model over another is largely due to the low number of species examined.

### Phylogenetic analysis of continuous reaction norms

We also analyzed the continuous-valued measures of four plasticity traits: slope of size plasticity in the ventral hind wing Cu1 eyespot and slopes of titer plasticity in juvenile hormone, ecdysone, and 20E, Using the Rphylopars (Goolsby et al., 2017) and ape (Paradis et al., 2004), packages for R (R Development Core Team, 2018). We estimated ancestral states separately for each trait, using the anc.recon function in Rphylopars. For each ancestral node, we used the 95% confidence interval to determine significance: if zero was excluded from the 95% C.I., the ancestral state was categorized as having a significantly non-zero slope.

### Data and materials availability

All data is available in the main text or the supplementary materials.

## Data Availability

All data generated or analysed during this study are included in the manuscript and supporting files. Source data files have been provided for Figures 1-4.

## Additional files

Transparent reporting form
