# Supplementary material for: Origin of the mechanism of phenotypic plasticity in satyrid butterfly eyespots
Source: eLife. 2020 Feb 11;9:e49544. doi: 10.7554/eLife.49544 (PMC7012602; doi:10.7554/eLife.49544)
Supplement: Figure 1—source data 1. — Table S1 - Species reared for comparative morphometrics, gene expression and hormonal measurements. Table S2 - F statistics, p-values from analysis of covariance for differences in Cu1 eyespot size between rearing temperatures (fixed factor) and assigned character state for phylogenetic analysis. [file elife-49544-fig1-data1.docx]

**Table S1.** **Species reared for comparative morphometrics, gene expression and hormonal measurements**

| **Species** | **Family/Nymphalid Subfamily** | **Spots/ Eyespots** | **Rearing temp. (**°C) | **Source of animals used in experiments** | **Climatic conditions (Köppen classification)** | **Reported Seasonality in Spot/Eyespot Size** |
| --- | --- | --- | --- | --- | --- | --- |
| *Junonia atlites* | **Nymphalid**ae/Nymphalinae | Eyespots | 20/30 | Malaysia | Tropical;  Equatorial humid (Af) | No |
| *Junonia coenia* | **Nymphalid**ae/Nymphalinae | Eyespots | 20/30 | USA | Subtropical | No |
| *Junonia iphita* | **Nymphalid**ae/Nymphalinae | Eyespots | 20/30 | Malaysia | Tropical;  Equatorial humid (Af) | No |
| *Junonia almana* | **Nymphalid**ae/Nymphalinae | Eyespots | 20/30 | Malaysia | Tropical;  Equatorial humid (Af) | Yes |
| *Doleschallia bisaltide* | **Nymphalid**ae/Nymphalinae | Eyespots | 20/30 | Malaysia | Tropical;  Equatorial humid (Af) | No |
| *Vanessa cardui* | **Nymphalid**ae/Nymphalinae | Eyespots | 17/27 | USA | Subtropical | No |
| *Vindula dejone* | **Nymphalid**ae/Heliconinae | Eyespots | 20/30 | Malaysia | Tropical;  Equatorial humid (Af) | No |
| *Cethosia cynae* | **Nymphalid**ae/Heliconinae | Eyespots | 20/30 | Singapore, Malaysia | Tropical;  Equatorial humid (Af) | No |
| *Bicyclus anynana* | **Nymphalid**ae/Satyrinae | Eyespots | 17/27 | Africa | Tropical;  Equatorial, winter dry (Aw) | Yes |
| *Morpho peleides* | **Nymphalid**ae/Morphinae | Eyespots | 17/27 | Costa Rica | Subtropical | No |
| *Danaus chryssipus* | **Nymphalid**ae/**Danainae** | Spots | 20/30 | Malaysia | Tropical; Equatorial humid (Af) | No |
| *Idea leuconoe* | **Nymphalid**ae/**Danainae** | Spots | 20/30 | Taiwan | Subtropical; Warm, humid, hot summers ^30^ | No |
| *Papilio polytes* | **Papilionidae - Outgroup** | Spots | 20/30 | Malaysia | Tropical; Equatorial humid (Af) | No |

Table S2. F statistics, p-values from analysis of covariance for differences in Cu1 eyespot size between rearing temperatures (fixed factor) and assigned character state for phylogenetic analysis. Wing size was used as a covariate. Rows highlighted in red indicate species where eyespot size decreases significantly with rearing temperature (negative slope). Species highlighted in green shows the opposite pattern (a significant positive slope). Character states of -1 = negative slope; 0=no plasticity; 1=positive slope.

| **Species** | **Family/**  **Subfamily** | **Factor** | **F stats** | **P value** | **DF (Factor, Error)** | **Slope of reaction norm** | **Character state for discrete values** |
| --- | --- | --- | --- | --- | --- | --- | --- |
| *Papilio polytes* | **Papilionidae** | temp. | 0.360 | 0.360 | 1,59 | 0.000 | 0 |
| *Danaus chrysippus* | **Danainae** | temp. | 0.318 | 0.585 | 1,59 | 0.008 | 0 |
| *Idea leucone* | **Danainae** | temp. | 10.073 | 0.031 | 1,59 | -0.004 | -1 |
| *Cethosia cyane* | Heliconinae | temp. | 0 | 0.096 | 1,59 | -0.004 | 0 |
| *Vindula dejone* | Heliconinae | temp. | 8.247 | 0.009 | 1,59 | -0.032 | -1 |
| *Vanessa cardui* | Nymphalinae | temp. | 15.056 | 0.001 | 1,59 | -0.016 | -1 |
| *Junonia almana* | Nymphalinae | temp. | 15.832 | <0.001 | 1,59 | -0.010 | -1 |
| *Junonia coenia* | Nymphalinae | temp. | 0.888 | 0.352 | 1,59 | 0.003 | 0 |
| *Junonia atlites* | Nymphalinae | temp. | 4.683 | 0.011 | 1,59 | -0.002 | -1 |
| *Junonia iphita* | Nymphalinae | temp. | 11.670 | 0.042 | 1,59 | -0.018 | -1 |
| *Doleschallia bisaltide* | Nymphalinae | temp. | 13.170 | 0.001 | 1,59 | -0.005 | -1 |
| *Bicyclus anynana* | Satyrinae | temp. | 42.769 | <0.001 | 1,59 | 0.057 | 1 |
| *Morpho peleides* | Morphinae | temp. | 0.765 | 0.393 | 1,19 | -0.007 | 0 |
