# Supplementary material for: Origin of the mechanism of phenotypic plasticity in satyrid butterfly eyespots
Source: eLife. 2020 Feb 11;9:e49544. doi: 10.7554/eLife.49544 (PMC7012602; doi:10.7554/eLife.49544)
Supplement: Figure 2—source data 1. [file elife-49544-fig2-data1.docx]

**Table S3 :** **F statistics, p-values from analysis of covariance for differences in 20E hormone titers between rearing temperatures (fixed factor) and assigned character states for phylogenetic analysis.** Wing size was used as a covariate. All data were log10 transformed to ensure linear allometries and comparable variances across temperature treatments. Rows highlighted in green indicate species where 20E titers increase significantly with rearing temperature (positive slope). Character state of 0=no plasticity; 1=positive slope.

| **Species** | **Factor** | **F stats** | **Slope of reaction norm** | **P value** | **DF (Factor, Error)** | **Character state for discrete values** |
| --- | --- | --- | --- | --- | --- | --- |
| *Papilio polytes* | Temperature | 0.004 | 126.087 | <0.0001 | 1,10 | 1 |
| *Danaus chrysippus* | Temperature | 1.234 | 12.424 | 0.293 | 1,10 | 0 |
| *Idea leucone* | Temperature | 0.000 | 176.991 | <0.0001 | 1,17 | 1 |
| *Cethosia cyane* | Temperature | 0.000 | 45.831 | 0.248 | 1,15 | 0 |
| *Vindula dejone* | Temperature | 10.199 | 289.120 | 0.005 | 1,17 | 1 |
| *Junonia almana* | Temperature | 5.830 | 24.134 | 0.034 | 1,25 | 1 |
| *Junonia atlites* | Temperature | 46.370 | 547.639 | <0.0001 | 1,11 | 1 |
| *Junonia iphita* | Temperature | 1.537 | 112.302 | 0.255 | 1,9 | 0 |
| *Doleschallia bisaltide* | Temperature | 31.481 | 192.753 | <0.0001 | 1,10 | 1 |
| *Bicyclus anynana* | Temperature | 34.304 | 185.891 | <0.0001 | 1,59 | 1 |
