# Supplementary material for: Origin of the mechanism of phenotypic plasticity in satyrid butterfly eyespots
Source: eLife. 2020 Feb 11;9:e49544. doi: 10.7554/eLife.49544 (PMC7012602; doi:10.7554/eLife.49544)
Supplement: Figure 3—source data 1. [file elife-49544-fig3-data1.docx]

**Table S4 : Mean body weight of wandering larvae, haemolymph volume and natural 20E titers at two different rearing temperatures; 20E and CucB injection volume**. (N=5 for measurement of means)

| **Species** | **Mean body weight** | **Mean total haemolymph volume** | **Total 20E**  **(in pg)** | | **20E injection** | | | **CucB injection** | | |
| --- | --- | --- | --- | --- | --- | --- | --- | --- | --- | --- |
|  |  |  | **20°C** | **30°C** | **Volume** | **Concentration (pg/μL)** | **Total (in pg)** | **Volume** | **Concentration (pg/μL)** | **Total (in pg)** |
| ***Idea leuconoe*** | 0.63g | 142 uL | 497500 | 709890 | 4 μl | 20000 | 80,000 | 3 μl | 20000 | 60000 |
| ***Vindula dejone*** | 0.45g | 88 uL | 191079 | 445505 | 3 μl | 10000 | 30000 | 3 μl | 10000 | 30000 |
| ***D. bisaltide*** | 0.49g | 95 uL | 170026 | 353142 | 3 μl | 7000 | 21000 | 3 μl | 10000 | 30000 |
| ***B. anynana*** | 0.19g | 61 uL | 85104 | 144165 | 3 μl | 2000 | 6000 | 2 μl | 5600 | 10,200 |
