# Supplementary material for: Origin of the mechanism of phenotypic plasticity in satyrid butterfly eyespots
Source: eLife. 2020 Feb 11;9:e49544. doi: 10.7554/eLife.49544 (PMC7012602; doi:10.7554/eLife.49544)
Supplement: Figure 4—source data 1. — See Figure 1—figure supplement 1 for node identities. [file elife-49544-fig4-data1.docx]

**Table S5 . Results of likelihood ratio tests and AIC comparisons.** See Fig. S1 for node identities.

| Character | Node | State | -lnL | ΔlnL | AICc | *w_i_* |
| --- | --- | --- | --- | --- | --- | --- |
| Size plasticity | 14 | Negative slope | 14.291 | 0 | 30.917 | 1.0 |
|  |  | Flat slope | 14.559 | 0.267 | 31.450 | 0.766 |
|  |  | Positive slope | 15.084 | 0.792 | 32.501 | 0.453 |
|  | 18 | Negative slope | 14.195 | 0 | 30.724 | 1.0 |
|  |  | Flat slope | 14.948 | 0.453 | 31.630 | 0.636 |
|  |  | Positive slope | 15.120 | 0.925 | 32.574 | 0.397 |
| 20 Hormone titre | 14 | Positive plasticity | 7.285 | 0 | 19.661 | 1.0 |
|  |  | No plasticity | 7.716 | 0.431 | 20.523 | 0.650 |
| EcR expression | 18 | EcR absent | 9.112 | 0 | 20.558 | 1.0 |
|  |  | EcR present | 9.604 | 0.491 | 21.571 | 0.612 |
