# Supplementary material for: Origin of the mechanism of phenotypic plasticity in satyrid butterfly eyespots
Source: eLife. 2020 Feb 11;9:e49544. doi: 10.7554/eLife.49544 (PMC7012602; doi:10.7554/eLife.49544)
Supplement: Peer review history

# Decision letter

**DOI:** 10.7554/eLife.49544.sa1

**Affiliations:** University of California, Los AngelesUnited States

In the interests of transparency, eLife publishes the most substantive revision requests and the accompanying author responses.

**Acceptance summary:**

Through its investigation of a well-studied trait in a remarkably charismatic and diverse group, this article has the potential to become a textbook example of the evolution of developmental plasticity. The article represents an impressive amount of research, and the approach taken, in which the authors worked backwards from the focal species to determine how the trait evolved was deemed "incredibly thought provoking and insightful" by reviewers. This article will be of benefit to *eLife* readers with interests in developmental biology, evo-devo, phenotypic plasticity, phenotypic evolution, and insect and butterfly evolution.

**Decision letter after peer review:**

[Editors’ note: the authors submitted for reconsideration following the decision after peer review. What follows is the decision letter after the first round of review.]

Thank you for submitting your work entitled "Origin of phenotypic plasticity in butterfly eyespots" for consideration by *eLife*. Your article has been reviewed by three peer reviewers, one of whom is a member of our Board of Reviewing Editors, and the evaluation has been overseen by a Senior Editor. The reviewers have opted to remain anonymous.

Our decision has been reached after consultation between the reviewers. Based on these discussions and the individual reviews below, we regret to inform you that your work will not be considered further for publication in *eLife*.

While all the reviewers appreciate the potential importance of the work completed by the authors, reviewer #3 in particular, a noted expert in this field, notes several fatal flaws in the study's design and the experimental implementation which prevent us from publishing the study in *eLife*. I strongly suggest that the authors review the comments of reviewer #3 before submitting their research to another journal.

Reviewer #2:

I thoroughly enjoyed reading this manuscript by Bhardwaj and colleagues. This is exciting, novel and truly unique research. The comparison of plastic responses using evo-devo responses across 12 species is unprecedented. The results suggest that the complex hormonal interactions that result in seasonal plasticity in *Bicyclus* butterflies originate in a step-wise manner – ecdysone sensitivity to temperature predates the plastic response, while ecdysone receptor expression in the eye spot area, and sensitivity to ecdysone are derived in the lineage with seasonal plasticity. These results have important implications for our understanding of the evolution of plasticity, and complex traits more generally. The figures in this manuscript are excellent visualizations of the main findings and patterns of phylogenetic variation. However, several clarifications are necessary with respect to the treatment of plasticity, and incorporation of literature outside of butterflies.

First, the manuscript as currently written is focused primarily on butterflies (e.g., from the second paragraph onwards), but the results are applicable to a much broader range of systems. It would be great if related examples from other systems could be incorporated in the Introduction and Discussion. For instance, work of JW Thornton on estrogen receptor evolution speaks to both regulators of plasticity (e.g., sex differences as developmental plasticity) and step-wise evolution of complex interactions between signaling molecules and receptors (e.g., preadaptation of ligands or receptors, see Thornton et al., 2003 Science, Thornton, 2001 PNAS, Bridgham et al., 2006 Science).

Second, the working definition of plasticity in this manuscript is fairly specific to polyphenisms, and other forms of evolved, developmental switches (sensu MJ West-Eberhard). This is important, because it affects some of the assumptions in the Introduction and the conclusions in the Discussion. For instance, the Introduction states that "we still have little understanding of how these complex adaptive responses originate and evolve across a phylogeny" and the Discussion concludes that "a warning that if many forms of adaptive plasticity are as specific and hard to evolve as the one documented in *B. anynana*, these exquisite adaptations to specific predictable fluctuating environments may in fact, lend the species vulnerable to extinction." These statements seem very much specific to "highly evolved" forms of plasticity like polyphenisms where developmental modules are switched on or off in response to an environmental cue. These statements seem less applicable to other forms of plasticity. For instance, Newman and Muller argued that plasticity – in terms of environmental sensitivity – is the ancestral state for most phenotypes, and that developmental canalization of originally environmentally induced body plans is derived (2000, "Epigenetic mechanisms of character origination"). In another case, consider trial-and-error learning as a type of plasticity – there are a plethora of studies looking at brain size and learning across a phylogeny, and these forms of plasticity are considerably less "fragile" with respect to performance in completely novel conditions. I suggest that the manuscript is framed around polyphenisms specifically, or evolved molecular interactions related to plasticity.

In terms of the methods, I have only one minor concern. I understand the challenges of rearing 12 species of butterflies, especially when they require different host plants and rearing conditions. However, the species were reared in three different locations, and *Bicyclus anynana* was reared in a unique location. It would be worth discussing in the Materials and methods the possible role of variation in rearing environment – is there any chance the differences across the species could be environmental rather than characteristics of the species?

Reviewer #3:

This manuscript takes a comparative look at temperature-controlled plasticity in the size of butterfly wing spot color patterns ("eyespots"). The study attempts to take a phylogenetic approach to infer the evolutionary history of different aspects of the eyespot temperature response mechanism. The authors look at the effect of rearing temperature on eyespot size across 13 species, which is an interesting and valuable exercise. They also look at temperature effects on titers of a hormone (20E) that affect color pattern polyphenism in some butterfly species, as well as expression of EcR, the presumed 20E receptor. Lastly, they look at the effects of injecting 20E, as well as a receptor antagonist, at one time point in four species. From these comparative datasets they make a phylogenetic argument that eyespot plasticity in one particular species occurred through gradual assembly of a novel hormone response mechanism. My overall thoughts are that the motivation of the work is very interesting, and there are some valuable data here. But there are also numerous fatal flaws in the study, in terms of experimental design and data quality (described below) that cause me to be highly skeptical about the authors' interpretation of the data.

Important conclusions of the paper are based mostly on negative results. My strongest criticism of this entire study, as it is with many other evo-devo papers that foray into phylogenetic character analysis, is that major conclusions are built on poorly controlled negative data. In fact, for almost every trait that is subject to phylogenetic analysis in this paper, I have serious concerns about problems with poor time series sampling, sampling at the wrong developmental stage, drawing homologies between species, etc. And the worry with all of these issues is negative data. Negative results from various species are counted as character states in a phylogenetic analysis which lead the authors to their conclusion about evolution of a developmental mechanism. But, as I describe below, there is strong reason to believe that a lack of time series data (and in one case, apparently wilful ignoring of published data) is biasing this work towards producing negative results.

Photoperiod is known to affect wing pattern plasticity, but was not controlled for between treatments and species. Rearing photoperiod is different between some species, and is simply not reported for many other species (I cannot find the information in the manuscript). This is a seriously confounding factor for the results in Figure 1 since it is known that photoperiod has a strong effect on plastic wing pattern traits in many butterfly species, both independent of, and in interaction with, temperature. Without photoperiod controls one cannot determine how much of a phenotypic response shown in Figure 1 is due to temperature vs. other environmental effects. To be publishable, for all species the photoperiod has to be the same for both temperature treatments, and photoperiod has to be reported in the paper.

There is no evidence that the *Papilio, Danaus*, and *Idea* "eyespots" share evolutionary or developmental homology with true nymphalid eyespots. I am unaware of any developmental, gene expression, or genetic data supporting homology of these color patterns with true eyespots. In my opinion these patterns are more likely to be derived from central or margin WntA patterning systems. They look just like patterns that are lost in WntA CRISPR knockouts in other species (Mazo-Vargas et al., 2017). If the authors' homology call is incorrect, then any data gained from analyzing these color patterns will completely distort the phylogenetic analysis. This potential problem is especially critical since these are outgroups.

EcR antibody stains are poor quality, lack proper controls, and are only for a single stage. The EcR immunostains in Figure 2 are important data for assembling the phylogenetic model. Unfortunately there are many problems with these data and they are not publishable as shown. First of all, there are no positive controls for the species that are scored as having no immunofluorescent signal. Do we know that antibody works in these species?

Second, there are no time expression series. Perhaps in the negative cases expression EcR a bit earlier or later. I am very perplexed by this lack of consideration for other time points besides larval wandering stage. For example, EcR expression has been published in *Junonia (Precis) coenia* eyespots at a later timepoint, in correlation with the eyespot transcription factor Distalless no less (Koch et al., 2003). But the authors completely ignore this in their model, and even score *J. coenia* EcR expression as negative in their phylogenetic analysis. I think this ignoring of different time points, and previously published data, is surprising and I hope not purposefully misleading. I also find it very odd that there are no *J. coenia* EcR immunostains in this paper, especially since one of the experts on the species (H. Nijhout) is a co-author on the paper. As I point out on multiple occasions in this review, the authors need to consider more than a single development stage to adequately address their research questions.

Third, some of the stains look poor quality. They don't look like spot expression to me, and the image size, framing, and resolution is poor so I can't tell of the signal is localized to the nucleus as one would expect for EcR. I would like to see a supplemental figure with whole-wing images for all species to see color pattern correlations more clearly and to rule out background fluorescence. I would like to see proper counterstaining. I would like to see positive controls for the EcR antibody in each species. I would like to see higher magnification images showing the purported "spot" patterns, as well as nuclear localizations. If possible, it would also make the data much stronger to see double stains to test for co-localization of EcR and some other eyespot marker. This would be especially important for some of the species in which the stains look more like diffuse smudges in the provided images.

Single-stage sampling for hormone titer comparisons doesn't make sense because critical periods are known to be different for different species. 20E titers only provided for a single timepoint in this study (wandering larvae), because this is the important stage for *B. anynana*. There are several important problems with this lack of temporal sampling. First of all, across insects the timing of hormone pulses and sensitive periods can be very specific and short-lived, and small changes in these pulses and periods are almost certainly significant for trait evolution. Therefore a single time point, generally, cannot serve as proxy for an entire endocrine process in a cross-species comparative study, at least without some sort of reference time series for each species. Second, this is almost certainly a problem in this study since it is known that the 20E pulse and sensitive period for color pattern polyphenism in *J. coenia* is at a later time point that is not even sampled in this paper (Rountree and Nijhout, 1995). I am concerned that by sampling only a single timepoint in all the species in this study, there may be important false negatives. Third, I think by not doing time series that there is simply a lot of interesting biology that is being missed. To me it is very interesting that both *B. anynana* and *J. coenia* express EcR in their eyespots (the latter observation is mostly ignored in this paper), yet have different sensitive periods and responses. Clearly there is more complex evolution going on here. I would like for this paper to talk more about this. Most importantly, though, they should have titer time series to have a complete, honest, and interesting story.

Staging all injection experiments at a single stage is not sufficient for comparative analysis because it is known that hormone-sensitive periods vary between species. All 20E and CucB injections were at a single stage (the wandering larva). This is problematic because many of the key conclusions of the paper are based on negative results from these injections. For many of the reasons outlined above, including previous experimental data from *J. coenia*, it is critical to do multiple timepoints. From these single time point injections we cannot rule out the possibility that the non-responsive species may simply be responsive at different time points. I am especially skeptical about *V. dejone*, which shows EcR expression in the eyespot and has a very strong eyespot temperature response. As I describe elsewhere, I am also skeptical that the black spots in *I. leucone* are homologous with *B. anyana* eyespots at all, so I am not sure how meaningful the results from this species are at all.

Odd inconsistency in injection controls. Shouldn't the "V" negative controls be the same between the 20E and CucB experiments? Yet they are extremely different, in some cases requiring completely different axis scales between experiments. In a few cases the differences between the different negative controls is much greater than the differences between the treatment and the negative control. The *B. anyana* results looks especially strange. Either I am missing something that needs to be explained better, or there was some problem with the experiments.

# Reply: Author response

**DOI:** 10.7554/eLife.49544.sa2

[Editors’ note: the authors resubmitted a revised version of the paper for consideration. What follows is the authors’ response to the first round of review.]

Reviewer #2:

I thoroughly enjoyed reading this manuscript by Bhardwaj and colleagues. This is exciting, novel and truly unique research. The comparison of plastic responses using evo-devo responses across 12 species is unprecedented. The results suggest that the complex hormonal interactions that result in seasonal plasticity in Bicyclus butterflies originate in a step-wise manner – ecdysone sensitivity to temperature predates the plastic response, while ecdysone receptor expression in the eye spot area, and sensitivity to ecdysone are derived in the lineage with seasonal plasticity. These results have important implications for our understanding of the evolution of plasticity, and complex traits more generally. The figures in this manuscript are excellent visualizations of the main findings and patterns of phylogenetic variation. However, several clarifications are necessary with respect to the treatment of plasticity, and incorporation of literature outside of butterflies.

First, the manuscript as currently written is focused primarily on butterflies (e.g., from the second paragraph onwards), but the results are applicable to a much broader range of systems. It would be great if related examples from other systems could be incorporated in the Introduction and Discussion. For instance, work of JW Thornton on estrogen receptor evolution speaks to both regulators of plasticity (e.g., sex differences as developmental plasticity) and step-wise evolution of complex interactions between signaling molecules and receptors (e.g., preadaptation of ligands or receptors, see Thornton et al., 2003 Science, Thornton, 2001 PNAS, Bridgham et al., 2006 Science).

Thank you. We have now modified the Introduction to include a more general paragraph about the origin of plasticity across different systems. We have refrained from including the references above (from Thornton et al.) because these references are more concerned with explaining how the steroid receptor family achieved its current diversity, and how receptor ligand systems co-evolve, something that we do not explore in the current work. As far as we know, the steroid receptor EcR is present across all butterfly species examined and in single copy.

Second, the working definition of plasticity in this manuscript is fairly specific to polyphenisms, and other forms of evolved, developmental switches (sensu MJ West-Eberhard). This is important, because it affects some of the assumptions in the Introduction and the conclusions in the Discussion. For instance, the Introduction states that "we still have little understanding of how these complex adaptive responses originate and evolve across a phylogeny" and the Discussion concludes that "a warning that if many forms of adaptive plasticity are as specific and hard to evolve as the one documented in B. anynana, these exquisite adaptations to specific predictable fluctuating environments may in fact, lend the species vulnerable to extinction." These statements seem very much specific to "highly evolved" forms of plasticity like polyphenisms where developmental modules are switched on or off in response to an environmental cue. These statements seem less applicable to other forms of plasticity. For instance, Newman and Muller argued that plasticity – in terms of environmental sensitivity – is the ancestral state for most phenotypes, and that developmental canalization of originally environmentally induced body plans is derived (2000, "Epigenetic mechanisms of character origination"). In another case, consider trial-and-error learning as a type of plasticity – there are a plethora of studies looking at brain size and learning across a phylogeny, and these forms of plasticity are considerably less "fragile" with respect to performance in completely novel conditions. I suggest that the manuscript is framed around polyphenisms specifically, or evolved molecular interactions related to plasticity.

Yes, we agree that there are different views on the origins of plasticity and whether or not plasticity is a derived or ancestral state for most organisms. In our opinion both views for the origin of plasticity are actually possible but it is important, in each case, to use the phylogenetic comparative method to explore ancestral states and direction of change, something that is rarely done, especially when examining how plastic systems evolve at the molecular, proximate level. We have included the paragraph below to introduce the distinct views that predominate the field of plasticity followed by explaining that our focus will be on examining the origin of the adaptive plasticity underlying wing pattern polyphenism in satyrids, represented by *Bicyclus anynana*:

“There are two disparate views regarding phenotypic plasticity. One regards plasticity as a derived adaptation to help organisms survive in variable environments (Bradshaw, 1965; de Jong, 2005) while the other views plasticity as the outcome of flexible, non-canalized, developmental processes, ancestrally present in most organisms, that helps them colonize or adapt to novel environments e.g., a pre-adaptation (Newman and Müller, 2000; West-Eberhard, 2003; Pigliucci, 2005; Laland et al., 2014). […] Here we focus our investigation on the mechanistic origins of an adaptive seasonal polyphenism where environmental cues experienced during development alter adult phenotypes to make them fit different seasonal recurrent environments, a highly evolved form of phenotypic plasticity.”

In terms of the methods, I have only one minor concern. I understand the challenges of rearing 12 species of butterflies, especially when they require different host plants and rearing conditions. However, the species were reared in three different locations, and Bicyclus anynana was reared in a unique location. It would be worth discussing in the Materials and methods the possible role of variation in rearing environment – is there any chance the differences across the species could be environmental rather than characteristics of the species?

Thank you. We have explained in our methods that these butterflies were reared in climate-controlled chambers across all 3 locations, where we monitored temperature and humidity across larval development. The environmental factors were consistent across locations, which implies that its effects are minimal in this study.

Reviewer #3:

This manuscript takes a comparative look at temperature-controlled plasticity in the size of butterfly wing spot color patterns ("eyespots"). The study attempts to take a phylogenetic approach to infer the evolutionary history of different aspects of the eyespot temperature response mechanism. The authors look at the effect of rearing temperature on eyespot size across 13 species, which is an interesting and valuable exercise. They also look at temperature effects on titers of a hormone (20E) that affect color pattern polyphenism in some butterfly species, as well as expression of EcR, the presumed 20E receptor. Lastly, they look at the effects of injecting 20E, as well as a receptor antagonist, at one time point in four species. From these comparative datasets they make a phylogenetic argument that eyespot plasticity in one particular species occurred through gradual assembly of a novel hormone response mechanism. My overall thoughts are that the motivation of the work is very interesting, and there are some valuable data here. But there are also numerous fatal flaws in the study, in terms of experimental design and data quality (described below) that cause me to be highly skeptical about the authors' interpretation of the data.

Important conclusions of the paper are based mostly on negative results. My strongest criticism of this entire study, as it is with many other evo-devo papers that foray into phylogenetic character analysis, is that major conclusions are built on poorly controlled negative data.

Thank you for your comment. Negative results (supported with appropriate controls) are as informative as positive results. The reviewer assumed that the data was built on poorly controlled negative data but that is not the case. We have included more information on how we controlled for these negative results in the Materials and methods section of the manuscript.

In fact, for almost every trait that is subject to phylogenetic analysis in this paper, I have serious concerns about problems with poor time series sampling, sampling at the wrong developmental stage, drawing homologies between species, etc. And the worry with all of these issues is negative data. Negative results from various species are counted as character states in a phylogenetic analysis which lead the authors to their conclusion about evolution of a developmental mechanism. But, as I describe below, there is strong reason to believe that a lack of time series data (and in one case, apparently willful ignoring of published data) is biasing this work towards producing negative results.

We think this criticism is unfair and misguided. It is also unclear what the reviewer thinks are “negative results”. We did not pursue any particular type of result when setting up to do this study. We merely report what we found. Furthermore, this work represents the most detailed study done to date in Lepidoptera that includes carefully sampled specimens at homologous stages of development. We initially used time-lapse photography to establish the Wr stage as the temperature sensitive stage for ventral Cu1 eyespot size plasticity in a model system (*Bicyclus anynana*) (Monteiro et al., 2015). Larvae were placed individually in transparent vertical containers with food, and photographed every 15 minutes. When the larvae left the food and climbed the container upwards (without returning) they were marked as having reached the beginning of the wandering stage. We performed the same time-lapse photography across all other species. Quantification of 20E titers, EcR stainings, and 20E injections were all performed based on this careful wandering stage staging to allow us to compare truly homologous points of development across all species. Most of this information was already detailed in the methods in the original submission but we have accentuated this further.

Photoperiod is known to affect wing pattern plasticity, but was not controlled for between treatments and species. Rearing photoperiod is different between some species, and is simply not reported for many other species (I cannot find the information in the manuscript). This is a seriously confounding factor for the results in Figure 1 since it is known that photoperiod has a strong effect on plastic wing pattern traits in many butterfly species, both independent of, and in interaction with, temperature. Without photoperiod controls one cannot determine how much of a phenotypic response shown in Figure 1 is due to temperature vs. other environmental effects. To be publishable, for all species the photoperiod has to be the same for both temperature treatments, and photoperiod has to be reported in the paper.

Thank you for pointing out this important omission. We have now modified the Materials and methods details to include this information:

“All species were reared at two temperatures separated by 10 degrees, at 70 or 80% RH and at 12:12h light: dark cycle. The only exeption was *Junonia coenia,* which was reared at 16:8h light: dark cycle. […] Humidity in these latter chambers was monitored using a PT2470 Hygrometer (Exoreptiles, Malaysia) and EL-USB-2 data loggers (Lascar Electronics, PA 16505, USA).”

There is no evidence that the Papilio, Danaus, and Idea "eyespots" share evolutionary or developmental homology with true nymphalid eyespots. I am unaware of any developmental, gene expression, or genetic data supporting homology of these color patterns with true eyespots. In my opinion these patterns are more likely to be derived from central or margin WntA patterning systems. They look just like patterns that are lost in WntA CRISPR knockouts in other species (Mazo-Vargas et al., 2017). If the authors' homology call is incorrect, then any data gained from analyzing these color patterns will completely distort the phylogenetic analysis. This potential problem is especially critical since these are outgroups.

Previous work suggested that eyespots originated from existing spots, perhaps like the ones shown in *Idea*, a Danainae (a basal-branching sub-family of the Nymphalidae) (Oliver et al., 2014). Because of this previous work, we decided to also examine species with simpler spots within and outside the Nymphalidae. The gene Spalt is a known marker for spots and eyespots across Nymphalidae and Pieridae (see Figure S2 of (Oliver et al., 2012), and (Stoehr et al., 2013)) and we have now modified Figure 2 to include Spalt co-stainings performed at the same time as EcR for all species. We observe that spots, localized at homologous positions as Cu1 eyespots, in *Papilio* and *Idea* express Spalt during development, just like eyespots. This shared expression pattern suggests (but does not conclusively show) that eyespots and spots may be homologous, and that spots may have preceded eyespots in evolution. Importantly, however, given that all outgroup species with spots did not express EcR in spots or in any region that might be homologous to eyespots, we concluded that the EcR expression pattern is likely to have originated in the sister lineage to the Danainae, which is also the lineage where eyespots originated. In *Danaus*, another Danainae, the wing margin spots are indeed likely to represent a wing margin patterning system that is impacted by WntA, which may not be homologous to either spots (like in *Idea*) or eyespots. Regardless, this result, contrary to what the reviewer suggests, does not “completely distort the phylogenetic analysis”. The inclusion of this species has no significant impact on our ancestral state reconstruction for the origin of EcR expression in regions that map to eyespot centers.

EcR antibody stains are poor quality, lack proper controls, and are only for a single stage. The EcR immunostains in Figure 2 are important data for assembling the phylogenetic model. Unfortunately there are many problems with these data and they are not publishable as shown. First of all, there are no positive controls for the species that are scored as having no immunofluorescent signal. Do we know that antibody works in these species?

We have now included a new Supplementary figure (Figure 2—figure supplement 2) to include positive controls of EcR expression in the large nuclei of the peripodial membrane as previously reported for EcR (Koch et al., 2003). In addition, we have added in the main Figure 2 Spalt (Sal) co-stainings because this gene marks future spot and eyespot centres. Sal expression was present across all species examined with spots/eyespots, irrespective of whether they expressed EcR in these pattern elements.

Second, there are no time expression series. Perhaps in the negative cases expression EcR a bit earlier or later. I am very perplexed by this lack of consideration for other time points besides larval wandering stage.

In previous work we established that the wandering stage is the critical sensitive stage of development when ventral Cu1 eyespot size is determined in *B. anynana* in response to temperature (Monteiro et al.,2015). This was our departure point for this comparative investigation. All animals used in our experiments were monitored during their entire development and we were able to confidently identify the same homologous wandering stage using time lapse photography across all species. Expanding the current work (with 13 species) to include sampling of EcR stainings at other time points would be prohibitive in terms of sampling effort and is not required to answer the question we posed at the beginning of this manuscript. Our work does not preclude future examinations of these alternative time points as candidate developmental stages that regulate plasticity in other species. Note, however, that often different cis-regulatory elements control the expression of the same gene across different body locations and even stages of development at homologous locations. A famous example of the latter are the different cis-elements of the gene *Distal-less* that initiate expression in early limbs of *Drosophila*, and then maintain this expression in later stages of limb development (McKay et al., 2009). This implies that the origin of novel cis-elements, or disruption of homologous and pre-existing cis-elements might be responsible for altering the expression pattern of genes over the course of development at homologous positions in the wing over evolutionary time. To avoid these confounding effects we restricted our examination of the molecular evolution of the mechanisms of plasticity in *Bicyclus anynana* by sampling strictly homologous tissues (ventral wings and Cu1 wing sectors) and time points across a phylogeny.

For example, EcR expression has been published in Junonia (Precis) coenia eyespots at a later timepoint, in correlation with the eyespot transcription factor Distalless no less (Koch et al., 2003). But the authors completely ignore this in their model, and even score J. coenia EcR expression as negative in their phylogenetic analysis. I think this ignoring of different time points, and previously published data, is surprising and I hope not purposefully misleading.

The reviewer is incorrect in assuming that we ignored this previous result. We are well aware of this previous study but for the reasons already outline above, we chose to focus our investigation on strictly homologous time periods of development across all species examined. Relative to *J. coenia*, previous work documented EcR expression in the peripodial membrane and along the trachea, but not in the focal eyespot central cells at the wandering stage of development (Koch et al., 2003). EcR is later observed in *J. coenia* eyespots during pupal development, which is a later stage and not the focus of this study. Given that close relatives such as *J. atlites* and *J. iphita* have EcR expression in the Cu1 ventral eyespot centers at the wandering stages of development, the most likely evolutionary explanation for the observed absence of EcR in eyespots in this species and also in *J. almana*, is the loss of this expression pattern at this stage of development. Note that this type of stage-specific EcR regulation has been documented before in *B. anynana* forewing ventral eyespots (in females only; Monteiro et al., 2015), where EcR is expressed in mid-larval stages and again in early pupal stages, but is absent at the wandering stage – allowing these eyespot centers to be insensitive to temperature. This allows these eyespots to retain the same size in both seasonal forms and function in sexual selection and/or predator avoidance year around. Again, we are neither ignoring other time-points (they are just beyond the scope of current study), nor purposefully misleading the readers.

I also find it very odd that there are no J. coenia EcR immunostains in this paper, especially since one of the experts on the species (H. Nijhout) is a co-author on the paper. As I point out on multiple occasions in this review, the authors need to consider more than a single development stage to adequately address their research questions.

While the reviewer is correct in pointing out that H. Nijhout is an expert and a co-author of the current study, we have carefully reviewed existing literature (performed by Koch and Nijhout) and used their specific EcR expression data from *J. coenia* (Koch et al., 2003) to conduct our ancestral state reconstructions.

Third, some of the stains look poor quality. They don't look like spot expression to me, and the image size, framing, and resolution is poor so I can't tell of the signal is localized to the nucleus as one would expect for EcR. I would like to see a supplemental figure with whole-wing images for all species to see color pattern correlations more clearly and to rule out background fluorescence. I would like to see proper counterstaining. I would like to see positive controls for the EcR antibody in each species. I would like to see higher magnification images showing the purported "spot" patterns, as well as nuclear localizations. If possible, it would also make the data much stronger to see double stains to test for co-localization of EcR and some other eyespot marker. This would be especially important for some of the species in which the stains look more like diffuse smudges in the provided images.

We have now included double stains for the positive control gene, Spalt, previously shown to mark spots and eyespots, as well as a supporting image for Figure 2, which includes positive controls for EcR stainings in the large nuclei of the peripodial membrane for all species except one (where no peripodial membrane was photographed at the time, and where the samples have since deteriorated). This species, however, had a positive EcR signal in the eyespot centers. The cells of the larval wing disc are very small and punctate nuclear stainings are very difficult to visualize. However, nuclear stainings in the polyploid nuclei of the peripodial membrane are easy to visualize and make appropriate positive controls for EcR stainings. The framing and the resolution of the images presented (which includes a full field of view taken with a 40X objective lens across all samples) was necessary for the proper confocal sectioning of just the ventral surface of the wing, the subject of the current investigation. Lower resolution would make it more difficult to isolate just the ventral signal from any signal present on the dorsal surface of the wing, which was not under investigation. Note that to provide this type of surface-specific data we performed left and right wing dissections separately to always be able to distinguish dorsal and ventral wing surfaces for each specimen examined.

Single-stage sampling for hormone titer comparisons doesn't make sense because critical periods are known to be different for different species. 20E titers only provided for a single timepoint in this study (wandering larvae), because this is the important stage for B. anynana. There are several important problems with this lack of temporal sampling. First of all, across insects the timing of hormone pulses and sensitive periods can be very specific and short-lived, and small changes in these pulses and periods are almost certainly significant for trait evolution. Therefore a single time point, generally, cannot serve as proxy for an entire endocrine process in a cross-species comparative study, at least without some sort of reference time series for each species. Second, this is almost certainly a problem in this study since it is known that the 20E pulse and sensitive period for color pattern polyphenism in J. coenia is at a later time point that is not even sampled in this paper (Rountree and Nijhout, 1995). I am concerned that by sampling only a single timepoint in all the species in this study, there may be important false negatives. Third, I think by not doing time series that there is simply a lot of interesting biology that is being missed. To me it is very interesting that both B. anynana and J. coenia express EcR in their eyespots (the latter observation is mostly ignored in this paper), yet have different sensitive periods and responses. Clearly there is more complex evolution going on here. I would like for this paper to talk more about this. Most importantly, though, they should have titer time series to have a complete, honest, and interesting story.

Yes, we agree with the reviewer that evolution of the critical time period for temperature sensitivity across species is a likely explanation for why other species do not exhibit the particular form of plasticity encountered in *B. anynana*. However, our goal was to try and examine how the specific pattern of plasticity in *B. anynana* evolved. We did not set out to explain how all patterns of plasticity that might be present across all these species evolved. This would be an unwieldy goal for a single study. Moreover, it is not possible for us to do additional time sampling at this stage due to experimental and funding limitations. We have modified our manuscript in specific sections to make our goals clearer and also mention that the critical time period for temperature sensitivity is likely evolving across species.

Staging all injection experiments at a single stage is not sufficient for comparative analysis because it is known that hormone-sensitive periods vary between species. All 20E and CucB injections were at a single stage (the wandering larva). This is problematic because many of the key conclusions of the paper are based on negative results from these injections. For many of the reasons outlined above, including previous experimental data from J. coenia, it is critical to do multiple timepoints. From these single time point injections we cannot rule out the possibility that the non-responsive species may simply be responsive at different time points. I am especially skeptical about V. dejone, which shows EcR expression in the eyespot and has a very strong eyespot temperature response. As I describe elsewhere, I am also skeptical that the black spots in I. leucone are homologous with B. anyana eyespots at all, so I am not sure how meaningful the results from this species are at all.

Yes, we agree with the reviewer that we cannot rule out the possibility that other species regulate their patterns of plasticity at different stages of development, but this is not what we set out to investigate. Again, we focused our investigation on the wandering stage, where there are definite temperature mediated differences in hormone titers and EcR signaling in *Bicyclus anynana*, the species around which we centered this investigation.

Odd inconsistency in injection controls. Shouldn't the "V" negative controls be the same between the 20E and CucB experiments? Yet they are extremely different, in some cases requiring completely different axis scales between experiments. In a few cases the differences between the different negative controls is much greater than the differences between the treatment and the negative control. The B. anyana results looks especially strange. Either I am missing something that needs to be explained better, or there was some problem with the experiments.

While “Vehicle” negative controls are indeed injected with the same ethanol-saline solution, we performed two batches of injections for the vehicle injected animals. One performed alongside the 20E injections, and another performed alongside the CucB injections. These two vehicle groups were kept separate for consistency. The small differences in “V” are due to the different batches of larvae used for the experiments.
